# Advanced Airway Practice Patterns and Out-of-Hospital Cardiac Arrest Outcomes

**DOI:** 10.1001/jamanetworkopen.2025.32334

**Published:** 2025-09-17

**Authors:** Michelle M. J. Nassal, Betty Y. Yang, Jane Hall, Jenny Shin, Christopher B. Gage, Jonathan R. Powell, Ashish R. Panchal, Andrew J. Latimer, Henry E. Wang, Thomas D. Rea, Nicholas J. Johnson

**Affiliations:** 1 Department of Emergency Medicine, The Ohio State University, Columbus; 2 Department of Emergency Medicine, University of Texas Southwestern Medical Center, Dallas; 3 Department of Emergency Medicine, University of Washington and Harborview Medical Center, Seattle; 4 Emergency Medical Services Division of Public Health, Seattle & King County, Seattle, Washington; 5 National Registry of Emergency Medical Technicians, Columbus, Ohio; 6 ImageTrend LLC, Eagan, Minnesota

## Abstract

**Importance:**

Although advanced airway (AA) practice patterns have varied over time, their association with out-of-hospital cardiac arrest (OHCA) outcomes is unknown.

**Objective:**

To determine the association between AA temporal practice patterns of emergency medical service (EMS) agencies and OHCA outcomes.

**Design, Setting, and Participants:**

This cross-sectional study used data from multicenter EMS agencies participating in the Cardiac Arrest Registry to Enhance Survival database. The study included adults (aged ≥18 years) with OHCA treated by EMS agencies that had 25 or more OHCA episodes annually from January 1, 2016, through December 31, 2022.

**Exposure:**

AA interventions included supraglottic airway (SGA) device use or endotracheal intubation (ETI). Patients were categorized into groups using the following EMS agency-level patterns defined by predominant AA use before and after 2019: (1) ongoing ETI, (2) ongoing SGA use (ongoing SGA), (3) transitioning from ETI to SGA use (ETI to SGA), or (4) transitioning from SGA use to ETI (SGA to ETI).

**Main Outcomes and Measures:**

Mixed-effects logistic regression models accounting for EMS agency clustering and adjusting for Utstein variables were used to evaluate the association between EMS agency AA practice patterns and OHCA outcomes including return of spontaneous circulation (ROSC) and survival. Subanalyses were also conducted for agencies in the lowest survival quartile. Odds ratios (ORs) are reported with 95% CIs.

**Results:**

This study included 350 216 patients with OHCA treated by 254 eligible EMS agencies. The 214 EMS agencies (n = 305 341 patients) with a predominant AA pattern were grouped as follows for temporal pattern analysis: ongoing ETI (n = 72 [33.6%]), ongoing SGA (n = 66 [30.8%]), ETI to SGA (n = 67 [31.3%]), or SGA to ETI (n = 9 [4.2%]). Patients were predominantly male (62.2%), with a median age of 64 (IQR, 52-76) years, and most (81.7%) presented with nonshockable rhythms. ROSC occurred in 30.8% of patients, and 10.4% of patients survived to hospital discharge. Predominant SGA use among EMS agencies increased from 65 agencies in 2016 to 113 in 2022. ROSC decreased in all 4 groups from before to after 2019 as follows: from 36.5% to 30.7% (OR, 0.80 [95% CI, 0.77-0.82]) for ongoing ETI, from 32.4% to 26.4% (OR, 0.75 [95% CI, 0.73-0.78]) for ongoing SGA, from 32.1% to 28.5% (OR, 0.88 [95% CI, 0.85-0.91]) for ETI to SGA, and from 36.7% to 33.3% (OR, 0.92 [95% CI, 0.83-1.03]) for SGA to ETI. For the 15 lower-performing agencies (n = 20 860 patients) that transitioned from ETI to SGA after vs before 2019, an association with higher ROSC (from 25.7% to 29.1%; OR, 1.16 [95% CI, 1.09-1.24]) and survival (from 5.6% to 6.3%; OR, 1.17 [95% CI, 1.04-1.32]) was observed.

**Conclusions and Relevance:**

In this cohort study, SGA use among EMS agencies increased over time. Although ROSC declined for all AA temporal practice patterns, the transition from ETI to SGA use at EMS agencies with lower baseline survival was associated with improved outcomes. Future studies are warranted to confirm these findings and to evaluate whether the observed associations are consistent across diverse populations.

## Key Points

**Question:**

Is there an association between advanced airway practice patterns and out-of-hospital cardiac arrest (OHCA) outcomes?

**Findings:**

In this cross-sectional study of 350 216 patients with OHCA treated by 254 emergency medical service (EMS) agencies, supraglottic airway (SGA) device use increased from 2016 through 2022. In EMS agencies with low survival rates before 2019, switching from endotracheal intubation to SGA device use was associated with improved outcomes.

**Meaning:**

These findings suggest that SGA device use by EMS agencies with lower survival rates may be associated with improved outcomes in patients with OHCA.

## Teaser

This cross-sectional study uses registry data to evaluate the association between advanced airway practice patterns of emergency medical service agencies and out-of-hospital cardiac arrest outcomes.

## Introduction

Out-of-hospital cardiac arrest (OHCA) is a leading cause of morbidity and mortality globally.^1^ Current American Heart Association guidelines recommend advanced airway (AA) placement during OHCA resuscitation to facilitate effective ventilation and improved outcomes.^2^ Prehospital AA management generally includes supraglottic airway (SGA) device placement or endotracheal intubation (ETI). Several factors can influence AA practice patterns, such as paramedic skill set, patient-related factors, and regional protocols or state laws. These factors have been associated with variability in AA practice patterns of emergency medical service (EMS) agencies across the US.^3^

Moreover, AA practice patterns have evolved.^4,5^ Initially, SGA devices were used primarily as rescue devices after failed ETI attempts.^6^ However, EMS agencies have increasingly adopted SGA devices as the first-line airway strategy in patients with OHCA. This transition may have been influenced by 2 large randomized clinical trials (PART and AIRWAYS-2) published in 2018 comparing SGA devices with ETI in patients with OHCA^7,8^ or by practice changes related to the COVID-19 pandemic.^9^

Despite the aforementioned changes, it remains unclear whether changes in AA practice patterns of EMS agencies affect outcomes following cardiac arrest. We sought to evaluate the temporal patterns of EMS agency AA use and determine their association with OHCA outcomes in the Cardiac Arrest Registry to Enhance Survival (CARES) network, the largest OHCA registry in the US.

## Methods

This cross-sectional study was approved by The Ohio State University Office of Responsible Research Practices as nonhuman participant research; therefore, it was exempt from institutional review board review, and the need for informed consent was waived. The study followed the Strengthening the Reporting of Observational Studies in Epidemiology (STROBE) reporting guideline.

### Study Design, Setting, and Participants

We performed a retrospective analysis using data from the CARES national database, which is a national registry of OHCA events managed by Emory University and the Centers for Disease Control and Prevention in Atlanta, Georgia. The CARES database includes data from more than 2300 EMS agencies in 46 US states, and it collects data through 3 sources: 911 dispatch centers, EMS clinicians, and receiving hospitals. Predefined required data elements describing OHCA characteristics, interventions, and outcomes are recorded in a web-based registry. Optional data elements, including prehospital AA use and attempts, may also be entered by individual EMS agencies. The full CARES data dictionary of required and optional data elements is available and was last updated in 2023.^10^ Race and ethnicity was determined by EMS agency records. These data were included because race is a known predictor in OHCA,^11^ and they are reported here as Black or African American, White, or other race or ethnicity (which includes American Indian or Alaska Native, Asian, Hispanic or Latino, Native Hawaiian or Other Pacific Islander, multiple races or ethnicities, or unknown race or ethnicity). The CARES database^12^ requires that participants achieve more than 99% data entry completeness and accuracy of required data elements to be included in the dataset.

We included all adults (≥18 years) with nontraumatic OHCA reported from January 1, 2016, through December 31, 2022. The CARES database only includes OHCA with documented resuscitation efforts (defined as EMS-performed cardiopulmonary resuscitation [CPR], any defibrillation, or both), including bystander-performed automated external defibrillator use.^13,14^ We limited analysis to EMS agencies that reported 25 or more OHCA resuscitations annually and answered 70% or more of AA optional data entries in the CARES database annually over this time period.

### Measures

AA interventions included ETI or SGA device use. SGA devices included the following, as entered by EMS agencies: i-gel (Intersurgical Ltd), King laryngeal tube (Ambu), Combitube esophageal-tracheal double lumen tube (Medtronic), Air-Q intubating laryngeal airway (AirLife), LMA (laryngeal mask airway), other SGA, or other SAD (supraglottic airway device).

We first evaluated whether there were EMS agency-level AA practice pattern changes over our cohort timespan. We used a random 10% sample of all eligible EMS agencies to determine the year with the most agency practice change. We identified 2019 as the inflection point in AA practice patterns (eFigure in Supplement 1). We set 2019 as the time point to compare pre and post EMS agency practice patterns. Excluding 2019 OHCAs, we subsequently classified EMS agencies into 4 groups based on their predominant airway practice pattern (>55%) before and after 2019: (1) predominant ETI use (ongoing ETI), (2) predominant SGA use (ongoing SGA), (3) transition from ETI to predominantly SGA (ETI to SGA), and (4) transition from SGA to predominantly ETI (SGA to ETI). We excluded EMS agencies that did not exhibit a predominant airway strategy (those with 1:1 SGA:ETI use) over this study period.

### Outcomes

The primary outcome of interest was return of spontaneous circulation (ROSC), because this is the primary outcome of interest for EMS agencies and is the outcome most temporally linked to the intervention. Secondary outcomes included survival to hospital discharge and survival with good neurologic recovery, which are often influenced by other aspects of care, including hospital and prognostication factors. Survival with good neurologic recovery was defined by the dichotomized Cerebral Performance Category (CPC) scale, in which a CPC score of 1 or 2 was classified as good neurologic recovery.^15^

### Statistical Analysis

We used the Cochran-Armitage test for trend to evaluate the number of EMS agencies using SGA as their predominant AA strategy over the time. To evaluate the association between EMS agency AA patterns and outcomes, we used mixed-effects logistic regression models, treating EMS agency as a random effect. Fixed effects included adjustments for Utstein variables, including age, sex, public location, race and ethnicity, witnessed status, initial rhythm, and bystander CPR.^16^ We evaluated model performance and selection using clinical judgment based on well-established OHCA variables and the Akaike information criterion.^16,17^ We report random effects in accountability by percentages with 95% CIs. AA practice patterns of EMS agencies before and after 2019 were treated as independent variables, then linearly combined to account for time (reference group: pre-2019 ETI).^18,19^ To evaluate AA practice changes in lower-performing agencies, we separately examined the subset of EMS agencies with the lowest pre-2019 survival quartile. Only patients with complete data were included in the mixed-effects logistic regression models. Significance levels were evaluated using 2-sided tests set at *P* < .05. Odds ratios (ORs) are reported with 95% CIs. We performed all analyses using Stata IC, version 17 (StataCorp LLC).

## Results

**Figure 1.**
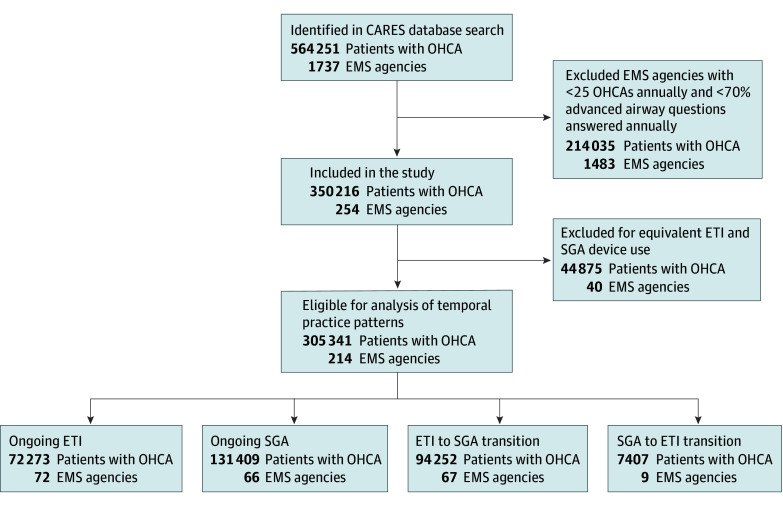
Study Flow Diagram All adults with out-of-hospital cardiac arrest (OHCA) in the Cardiac Arrest Registry to Enhance Survival (CARES) database from 2016 through 2022 were considered for inclusion. The predominant advanced airway strategy of each emergency medical services (EMS) agency before and after 2019 was categorized into 1 of 4 observed practice patterns: ongoing endotracheal intubation (ETI), ongoing supraglottic airway (SGA) device use, transition from ETI to SGA use (ETI to SGA), or transition from SGA use to ETI (SGA to ETI).

For the period from 2016 through 2022, 82.2% of all EMS agencies answered 70% or more AA questions annually and 17.1% of EMS agencies treated greater than 25 OHCAs annually, resulting in 254 EMS agencies and 350 216 patients with OHCA eligible for inclusion in this study (Figure 1). Of the 254 eligible EMS agencies, 40 were excluded from the temporal pattern analysis because they did not have a predominant AA strategy throughout the years.

**Figure 2.**
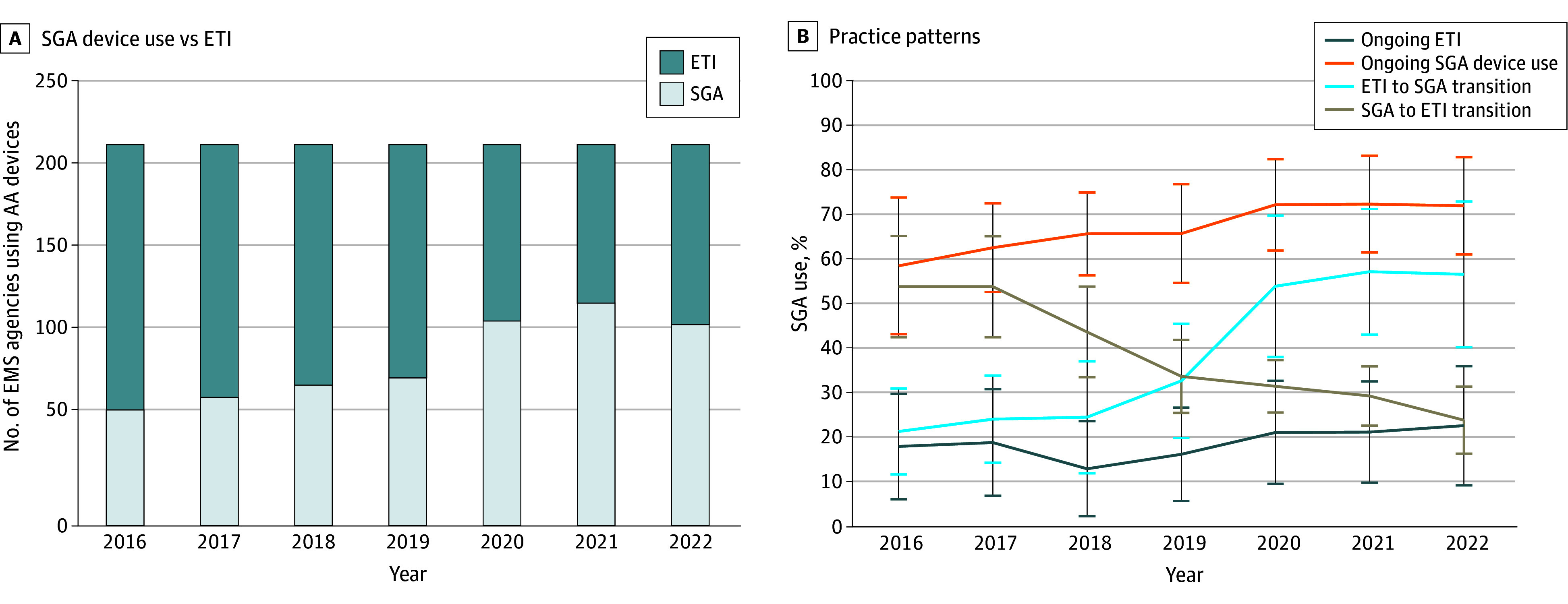
Predominant Advanced Airway (AA) Practice Patterns of Emergency Medical Service (EMS) Agencies, 2016-2022 (A) The majority of EMS agencies used endotracheal intubation (ETI) as their predominant AA strategy during out-of-hospital cardiac arrest in 2016. Supraglottic airway (SGA) device use became predominant among EMS agencies over time (*P* for trend = .048). (B) Line graphs show the 4 observed practice patterns from 2016 to 2022: ongoing ETI, ongoing SGA use, transition from ETI to SGA use (ETI to SGA), or transition from SGA use to ETI (SGA to ETI). There was a significant increase in SGA use in EMS agencies transitioning from ETI to SGA use (*P* for trend <.001).

We classified 214 EMS agencies (n = 305 341 patients with OHCA) into 1 of 4 temporal AA practice patterns as follows: (1) ongoing ETI, with 72 agencies (33.6%) and 72 273 patients (23.7%) with OHCA; (2) ongoing SGA use, with 66 agencies (30.8%) and 131 409 patients (43.0%); (3) transitioning from ETI to SGA use, with 67 agencies (31.3%) and 94 252 patients (30.9%); and (4) transitioning from SGA use to ETI, with 9 agencies (4.2%) and 7407 patients (2.4%). In 2016, the majority of EMS agencies (n = 149 of 214 [69.6%]) used ETI as their predominant AA strategy during OHCA resuscitation (Figure 2). Over time, SGA use increased such that the majority of EMS agencies (n = 113 [52.8%]) used these devices as their predominant AA strategy in 2022 compared with 2016 (*P* for trend = .048).

Of the 305 341 patients with OHCA included in the temporal practice pattern analysis, 62.2% were male and 37.8% were female. Their median age was 64 years (IQR, 52-76 years). The majority of patients (81.7%) presented with nonshockable rhythms. These characteristics were similar across all 4 EMS agency practice pattern cohorts (Table 1). A total of 23.3% of patients were Black or African American, 48.8% were White, and 27.9% were of other race or ethnicity. ROSC occurred in 30.8% of patients; 10.4% survived to hospital discharge, and 8.3% survived with good neurologic recovery. ROSC, survival to hospital discharge, and survival with good neurologic recovery appeared lower in all groups by EMS agency practice patterns after 2019 (eTable 1 in Supplement 1).

**Table 1.** Demographics of Groups in Practice Pattern Analysis^a^

Characteristic	Patients with OHCA (n = 305 341)	Agency practice pattern^b^			
		Ongoing ETI (n = 72 273)	Ongoing SGA (n = 131 409)	ETI to SGA (n = 94 252)	SGA to ETI (n = 7407)
Age, median (IQR), y	64 (52-76)	65 (53-76)	64 (53-75)	64 (52-76)	67 (56-77)
Missing	130 (<0.001)	8 (<0.001)	99 (0.07)	23 (<0.001)	0
Sex					
Male	189 907 (62.2)	45 780 (63.3)	81 000 (61.6)	58 319 (61.9)	4808 (64.9)
Female	115 431 (37.8)	26 492 (36.7)	50 408 (38.4)	35 932 (38.1)	2599 (35.1)
Missing	3 (<0.001)	1 (<0.001)	1 (<0.001)	1 (<0.001)	0
Race and ethnicity^c^					
Black or African American	71 265 (23.3)	9378 (13.0)	42 013 (32.0)	19 162 (20.3)	712 (9.6)
White	149 008 (48.8)	34 025 (47.1)	63 012 (48.0)	46 368 (49.2)	5603 (75.6)
Other race or ethnicity^d^	85 068 (27.9)	28 870 (39.9)	26 384 (20.1)	28 722 (30.5)	1092 (14.7)
Location of OHCA					
Home or residence	215 971 (70.7)	51 783 (71.7)	93 393 (71.1)	65 213 (69.2)	5582 (75.4)
Missing	2 (<0.001)	2 (<0.001)	0	0	0
Witnessed status					
Unwitnessed	155 034 (50.8)	36 357 (50.3)	66 259 (50.4)	49 152 (52.2)	3266 (44.1)
Bystander witnessed^e^	111 595 (36.6)	27 279 (37.7)	47 843 (36.4)	33 275 (35.3)	3198 (43.2)
911 responder	38 706 (12.7)	8636 (11.9)	17 307 (13.2)	11 820 (12.5)	943 (12.7)
Missing	6 (<0.001)	1 (<0.001)	0	5 (<0.001)	0
Initial rhythm					
Shockable	55 783 (18.3)	14 206 (19.7)	23 184 (17.6)	16 521 (17.5)	1827 (24.7)
Nonshockable	249 518 (81.7)	58 058 (80.3)	108 208 (82.4)	77 717 (82.5)	5580 (75.3)
Missing	40 (<0.001)	9 (<0.001)	17 (<0.001)	14 (<0.001)	0
Bystander CPR, No./total No.^f^	92 148/226 451 (40.7)	24 145/54 466 (44.3)	38 054/96 298 (39.5)	26 921/69 979 (38.5)	3028/5704 (53.1)

Abbreviations: CPR, cardiopulmonary resuscitation; ETI, endotracheal intubation; OHCA, out-of-hospital cardiac arrest; SGA, supraglottic airway.

^a^ Unless indicated otherwise, values are presented as No. (%) of patients.

^b^ Defined for the period before and after 2019 as follows: predominant ETI use (ongoing ETI), predominant SGA use (ongoing SGA), transition from ETI to predominantly SGA use (ETI to SGA), and transition from SGA use to predominantly ETI (SGA to ETI).

^c^ Determined by emergency medical service agency records.

^d^ Includes American Indian or Alaska Native, Asian, Hispanic or Latino, Native Hawaiian or Other Pacific Islander, multiple races or ethnicities, or unknown race or ethnicity.

^e^ Bystander CPR-eligible cases excluded all nontraumatic resuscitations that occurred in a health care facility, nursing home, physician office or clinic, and 911 responder–witnessed cardiac arrests.

^f^ Values are listed as No. of patients who received bystander CPR/Total No. (%) of patients with OHCA eligible for bystander CPR.

Rates of ROSC decreased from before to after 2019 as follows: from 36.5% to 30.7% for ongoing ETI, from 32.4% to 26.4% for ongoing SGA, from 32.1% to 28.5% for ETI to SGA, and from 36.7% to 33.3% for SGA to ETI. After adjusting for Utstein variables, 3 of the 4 EMS agency practice patterns were associated with decreased odds of ROSC after 2019: ongoing ETI (OR, 0.80 [95% CI, 0.77-0.82]), ongoing SGA (OR, 0.75 [95% CI, 0.73-0.78]), ETI to SGA (OR, 0.88 [95% CI, 0.85-0.91]), and SGA to ETI (OR, 0.92 [95% CI, 0.83-1.03]) (Table 2). Ongoing ETI and ongoing SGA practice patterns were also associated with lower odds of survival to hospital discharge and survival with good neurologic recovery in the post-2019 period compared with the pre-2019 period (Table 2). In contrast, a transition from ETI to SGA was not associated with lower odds of survival to hospital discharge or survival with good neurologic recovery after 2019 (Table 2).

**Table 2.** Associations Between EMS Agency Airway Practice Patterns and OHCA Outcomes

Agency practice pattern^a^	No. (%) of EMS agencies (n = 214)	No. of patients with OHCA	Outcome^b^					
			Return of spontaneous circulation		Survival to hospital discharge		Survival with good neurologic recovery^c^	
			AOR (95% CI)	*P* value	AOR (95% CI)	*P* value	AOR (95% CI)	*P* value
Ongoing ETI	72 (33.6)	62 437	0.80 (0.77-0.82)	<.001	0.91 (0.86-0.96)	<.001	0.86 (0.81-0.92)	<.001
Ongoing SGA	66 (30.8)	113 353	0.75 (0.73-0.78)	<.001	0.91 (0.88-0.95)	<.001	0.90 (0.86-0.94)	<.001
ETI to SGA	67 (31.3)	81 607	0.88 (0.85-0.91)	<.001	1.00 (0.96-1.06)	.62	0.97 (0.91-1.00)	.25
SGA to ETI	9 (4.2)	6375	0.92 (0.83-1.03)	.15	0.86 (0.73-1.01)	.07	0.80 (0.67-0.95)	.01

Abbreviations: AOR, adjusted odds ratio; EMS, emergency medical service; ETI, endotracheal intubation; OHCA, out-of-hospital cardiac arrest; SGA, supraglottic airway.

^a^ Defined for the period before and after 2019 as follows: predominant ETI use (ongoing ETI), predominant SGA use (ongoing SGA), transition from ETI to predominantly SGA use (ETI to SGA), and transition from SGA use to predominantly ETI (SGA to ETI).

^b^ Mixed-effects logistic regression model AORs are provided.

^c^ Defined as a Cerebral Performance Category scale score of 1 or 2.

EMS agency clustering accounted for approximately 33.9% (95% CI, 30.5%-37.6%) of the variability in ROSC outcome, 32.6% (95% CI, 28.9%-36.8%) of the variability in survival outcome, and 37.7% (95% CI, 33.6%-42.4%) of the variability in survival with good neurologic recovery outcome. Utstein variables associated with ROSC are presented in eTable 2 in Supplement 1. In summary, the covariates with the highest odds ratios for ROSC were EMS-witnessed OHCA (OR, 2.75 [95% CI, 2.67-2.82]), bystander-witnessed OHCA (OR, 2.43 [95% CI, 2.38-2.48]), and shockable rhythms (OR, 2.20 [95% CI, 2.14-2.24]).

When only the 52 EMS agencies (n = 63 877 patients) in the lowest survival quartile were considered, ongoing ETI and ongoing SGA use were associated with lower odds of ROSC and survival to hospital discharge similar to the overall cohort (Table 3). However, unlike the overall cohort, ongoing SGA use was associated with slightly higher odds of survival with good neurologic recovery (OR, 1.16 [95% CI, 1.02-1.33]). The 15 EMS agencies (n = 20 860 patients) in the lowest survival quartile that transitioned from ETI to SGA had higher odds of ROSC (from 25.7% to 29.1%; OR, 1.16 [95% CI, 1.09-1.24]) and survival to hospital discharge (from 5.6% to 6.3%; OR, 1.17 [95% CI, 1.04-1.32]), but not survival with good neurologic recovery (from 3.8% to 4.1%; OR, 1.12 [95% CI, 0.97-1.30]) (Table 3). EMS agencies in the top 3 survival quartiles had ORs for outcomes similar to those for the overall cohort (eTable 3 in Supplement 1). Unadjusted OHCA outcomes in the bottom quartile are presented in eTable 4 in Supplement 1.

**Table 3.** Associations Between EMS Agency Practice Patterns and OHCA Outcomes in the Lowest Survival Quartile

Agency practice pattern^a^	No. (%) of EMS agencies (n = 52)	No. (%) of patients with OHCA (n = 63 877)	Outcome^b^					
			Return of spontaneous circulation		Survival to hospital discharge		Survival with good neurologic recovery^c^	
			AOR (95% CI)	*P* value	AOR (95% CI)	*P* value	AOR (95% CI)	*P* value
Ongoing ETI	18 (34.6)	17 758 (27.8)	0.76 (0.72-0.82)	<.001	0.93 (0.82-1.05)	.26	0.99 (0.86-1.14)	.87
Ongoing SGA	18 (34.6)	24 996 (39.1)	0.91 (0.86-0.97)	.003	1.11 (1.0-1.24)	.06	1.16 (1.02-1.33)	.028
ETI to SGA	15 (28.8)	20 860 (32.7)	1.16 (1.09-1.24)	<.001	1.17 (1.04-1.32)	.011	1.12 (0.97-1.30)	.12
SGA to ETI	1 (1.9)	263 (0.4)	0.80 (0.46-1.39)	.43	0.52 (0.16-1.72)	.28	0.39 (0.10-1.49)	.17

Abbreviations: AOR, adjusted odds ratio; EMS, emergency medical service; ETI, endotracheal intubation; OHCA, out-of-hospital cardiac arrest; SGA, supraglottic airway.

^a^ Defined as predominant ETI use before and after 2019 (ongoing ETI), predominant SGA device use before and after 2019 (ongoing SGA), transition from ETI to predominantly SGA use (ETI to SGA), and transition from SGA use to predominantly ETI (SGA to ETI).

^b^ Mixed-effects logistic regression model AORs are provided.

^c^ Defined as a Cerebral Performance Category scale score of 1 or 2.

## Discussion

To our knowledge, this is the first evaluation of EMS agency AA temporal practice patterns and their associations with OHCA outcomes. EMS agency practice patterns varied from 2016 through 2022, with increasing SGA use and decreasing ETI use overall. A temporal decline in outcomes was observed in specific EMS agency practice patterns after 2019, likely corresponding in part to the COVID-19 pandemic.^20^ Interestingly, in lower-performing EMS agencies with lower survival rates that transitioned from ETI to SGA use, we observed an increased odds of ROSC and survival after 2019. Transitioning from ETI to SGA use was associated with improved outcomes, which suggest that transitions from ETI to SGA may have mitigated any worsening of outcomes observed in other EMS practice pattern cohorts.

Multiple factors, including published trials, the COVID-19 pandemic, and ease of SGA insertion, have likely contributed to increased SGA use.^21^ Airway management strategies, including bag-valve-mask ventilation and AA strategies, have shown variable associations with OHCA outcomes.^22,23^ Two large randomized clinical trials comparing AA strategies, PART and AIRWAYS-2, reported different findings: The PART trial investigators found a 72-hour survival benefit with SGA use, whereas the AIRWAYS-2 researchers found no difference in 30-day survival with good neurologic function.^7,8^ Secondary analysis of the PART trial did not show variability in ventilation rates, end-tidal carbon dioxide trends, or rates of first-pass success as the mechanism underlying improved OHCA survival.^21,24,25^ Interestingly, we and others observed increasing SGA use in the subsequent years following these 2 publications.^4,5^ Transitioning to predominant SGA use also coincided with the COVID-19 pandemic, which also influenced AA protocols and preferences.^9^ Beyond trials and pandemic protocols, SGA use likely increased because of the ease of reliable AA placement during OHCA.^8^ Based on these studies and other evidence, resuscitation guidelines indicate that AA strategies should consider clinician and agency experience.^2^

In general, we observed worse OHCA outcomes after 2019, likely related to practice changes around the COVID-19 pandemic, including less bystander CPR and substantial delays in patient care.^9,26^ We observed a decline in outcomes among agencies that maintained SGA or ETI strategies throughout, suggesting that neither approach completely mitigated the challenges related to COVID-19. We did, however, observe that those agencies that transitioned from ETI to SGA, especially lower-performing agencies, appeared to experience relatively less adverse survival impact during the post-2019 period. One interpretation is that lower-performing agencies may be better suited to use an SGA approach, given it is technically more straightforward and may not distract from other essential aspects of resuscitation as ETI might. This may provide the opportunity for relative outcome improvement. Although it is possible that SGA use itself mitigated against this decrease in survival, other factors such as EMS training in switching to a different predominant airway strategy, COVID-19 pandemic–related changes, or other unmeasured changes contributed to the observed associations in these analyses. We emphasize that both AA use strategies are important skills for EMS practitioners and that SGA use, which requires less technical skill, may be beneficial in certain EMS agencies or circumstances.

These data have important clinical implications. Approximately one-third (76 of 214 [35.5%]) of EMS agencies changed their primary AA approach during this study period, highlighting the dynamic nature of prehospital airway management currently. The choice of AA approach is complex and may influence clinical outcomes, as evidenced by differential temporal trends in clinical outcomes associated with AA strategy. These data suggest that it is reasonable to consider SGA strategies in agencies with poor survival rates or potentially lower volumes of AA use.^27,28^ SGA use may serve as an efficient airway management strategy to provide effective ventilations early in the resuscitation, because bag-valve-mask ventilation is often insufficient and can contribute to poor outcomes.^29^ There may be other characteristics for which airway practice change may predict outcome improvement, and a better understanding of these characteristics can help improve system decisions and ultimately clinical outcomes. Unfortunately, detailed qualitative data describing EMS agencies’ practice pattern changes were not available for this analysis.

### Limitations

Important limitations must be considered. Given the retrospective nature of this study, we cannot speculate on causation, only on association. We highlight that this study evaluated broad EMS agency practice pattern associations with OHCA outcomes, not the AA methods used in each cardiac arrest. Others have evaluated use of the King laryngeal tube vs the i-gel SGA in the CARES network, finding that i-gel was associated with improved neurologic outcomes.^30^ Although many EMS agencies participate consistently in the CARES network, the registry accounts for approximately 53% of the US population, although it is thought to be broadly representative.^10^ Further, this study only included a smaller portion of CARES agencies that attempt resuscitation in at least 25 OHCAs annually. Some states have notably less rural EMS agency participation.^31^ Generalizability to rural agencies or EMS agencies that less frequently provide resuscitations is not clear. There were far fewer agencies that transitioned from SGA to ETI compared with the other 3 practice pattern groups, limiting the ability to make inferential assessments. The CARES database has robust data processing for its required data entry items; however, AA questions are optional supplemental data entries. We included patients treated by EMS agencies with AA supplemental questions answered 70% of the time or more, which was 82.2% of all patients over this period. Finally, multiple AA devices could be listed by an EMS agency, but the CARES database does not detail device placement order. The CARES database also does not detail EMS agencies’ new device implementation periods; however, we attempted to mitigate this limitation by excluding 2019 OHCAs in EMS agency practice pattern classifications.

## Conclusions

In this cross-sectional study, AA practice patterns of EMS agencies during OHCA varied from 2016 through 2022, with increasing SGA use. Although ROSC, survival to hospital discharge, and survival with good neurologic recovery declined overall, transition from ETI to SGA use in lower-performing agencies was associated with improved outcomes. Future studies are needed to validate these findings and to evaluate whether the observed associations are consistent across diverse populations.

## Notes

Supplement 1. **eFigure.** Random Sampling of Emergency Medical Service (EMS) Agencies’ Endotracheal Tube (ETT) and Supraglottic Airway (SGA) Device Use, 2016-2022 **eTable 1.** Outcomes for Each Cohort **eTable 2.** Mixed-Effects Model for Return of Spontaneous Circulation Adjusted for Utstein Variables as Fixed Effects and Emergency Medical Services Agency as a Random Effect **eTable 3.** Top 3 Survival Quartiles Mixed-Effects Models for Out-of-Hospital Cardiac Arrest Outcomes **eTable 4.** Bottom Quartile of Unadjusted Out-of-Hospital Cardiac Arrest Outcomes

Supplement 2.
Data Sharing Statement
