# Supplementary material for: Advanced Airway Practice Patterns and Out-of-Hospital Cardiac Arrest Outcomes
Source: JAMA Netw Open. 2025 Sep 17;8(9):e2532334. doi: 10.1001/jamanetworkopen.2025.32334 (PMC12444570; doi:10.1001/jamanetworkopen.2025.32334)
Supplement: Supplement 1. — eFigure. Random Sampling of Emergency Medical Service (EMS) Agencies’ Endotracheal Tube (ETT) and Supraglottic Airway (SGA) Device Use, 2016-2022 eTable 1. Outcomes for Each Cohort eTable 2. Mixed-Effects Model for Return of Spontaneous Circulation Adjusted for Utstein Variables as Fixed Effects and Emergency Medical Services Agency as a Random Effect eTable 3. Top 3 Survival Quartiles Mixed-Effects Models for Out-of-Hospital Cardiac Arrest Outcomes eTable 4. Bottom Quartile of Unadjusted Out-of-Hospital Cardiac Arrest Outcomes [file jamanetwopen-e2532334-s001.pdf]

# Supplemental Online Content

Nassal MMJ, Yang BY, Hall J, et al. Advanced airway practice patterns and out-of-hospital cardiac arrest outcomes. *JAMA Netw Open*. 2025;8(9):e2532334. doi:10.1001/jamanetworkopen.2025.32334

**eFigure.** Random Sampling of Emergency Medical Service (EMS) Agencies' Endotracheal Tube (ETT) and Supraglottic Airway (SGA) Device Use, 2016-2022

**eTable 1.** Outcomes for Each Cohort

**eTable 2.** Mixed-Effects Model for Return of Spontaneous Circulation Adjusted for Utstein Variables as Fixed Effects and Emergency Medical Services Agency as a Random Effect

**eTable 3.** Top 3 Survival Quartiles Mixed-Effects Models for Out-of-Hospital Cardiac Arrest Outcomes

**eTable 4.** Bottom Quartile of Unadjusted Out-of-Hospital Cardiac Arrest Outcomes

This supplemental material has been provided by the authors to give readers additional information about their work.

© 2025 Nassal MMJ et al. *JAMA Network Open*.

**eFigure.** Random Sampling of Emergency Medical Service (EMS) Agencies' Endotracheal Tube (ETT) and Supraglottic Airway (SGA) Device Use, 2016-2022

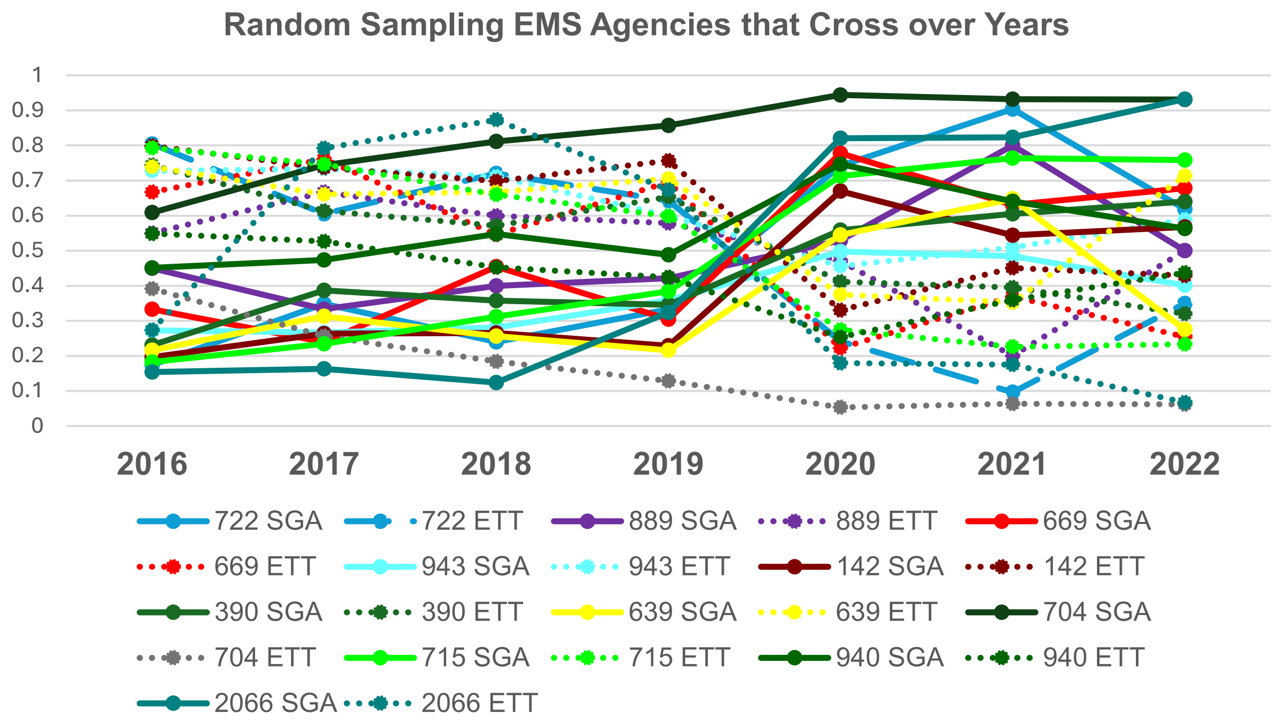


EMS Agencies percentage use of ETT (#ETT/total advanced airways in dashed lines) and SGA (#SGA/total advanced airways in dotted lines) were plotted over time. We randomly sampled 10% of all eligible EMS agencies and graphed 10 agencies of those that showed changes in advanced airway use over time. We also graphed one EMS agency out of the random sample that did not show a change in advanced airway use over time.

© 2025 Nassal MMJ et al. *JAMA Network Open*.

**eTable 1.** Outcomes for Each Cohort

| EMS Agency AA Practice Pattern | ROSC | | Survival | | Survival with Favorable Neurologic Recovery | |
|---|---|---|---|---|---|---|
| | Before 2019 | After 2019 | Before 2019 | After 2019 | Before 2019 | After 2019 |
| **Entire Cohort** | 39344 / 118288 (33.3%) | 41004 / 145484 (28.2%) | 12932 / 118288 (11.0%) | 14141 / 145484 (9.7%) | 10598 / 118288 (9.0%) | 11279 / 145484 (7.8%) |
| **Ongoing ETI 72 EMS Agencies** | 10039 / 27844 (36.5%) | 10615 / 34593 (30.7%) | 3297 / 27844 (11.8%) | 3588 / 34430 (10.4%) | 2887 / 27844 (10.4%) | 3051 / 34430 (8.8%) |
| **Ongoing SGA 66 EMS Agencies** | 16472 / 50776 (32.4%) | 16486 / 62577 (26.4%) | 5487 / 50772 (10.8%) | 5922 / 62577 (9.5%) | 4327 / 50772 (8.5%) | 4588 / 62577 (7.3%) |
| **ETI-to-SGA 67 EMS Agencies** | 11777 / 36789 (32.1%) | 12739 / 44818 (28.5%) | 3763 / 36789 (10.2%) | 4265 / 44818 (9.5%) | 3034 / 36789 (8.3%) | 3324 / 44818 (7.4%) |
| **SGA-to-ETI 9 EMS Agencies** | 1056 / 2879 (36.7%) | 1164 / 3496 (33.3%) | 385 / 2879 (13.4%) | 366 / 3496 (10.5%) | 350 / 2879 (12.5%) | 316 / 3496 (9.0%) |

Return of spontaneous circulation (ROSC). Endotracheal Intubation (ETI), Supraglottic Airway (SGA)

© 2025 Nassal MMJ et al. *JAMA Network Open*.

**eTable 2.** Mixed-Effects Model for Return of Spontaneous Circulation Adjusted for Utstein Variables as Fixed Effects and Emergency Medical Services Agency as a Random Effect

| Variable | OR (CI) | P-value |
|---|---|---|
| Age | 1.00 (1.00-1.00) | <0.001 |
| Male | 0.75 (0.73-0.76) | <0.001 |
| Race | | |
| White | Ref | |
| African-American | 0.85 (0.83-0.87) | <0.001 |
| Other race | 0.95 (0.93-0.98) | <0.001 |
| Location | | |
| Nursing home | 1.11 (1.08-1.14) | <0.001 |
| Public | 1.29 (1.26-1.33) | <0.001 |
| Witnessed Status | | |
| Bystander witnessed | 2.43 (2.38-2.48) | <0.001 |
| EMS Witnessed | 2.75 (2.67-2.82) | <0.001 |
| Shockable Rhythm | 2.20 (2.14-2.24) | <0.001 |
| Bystander CPR | 1.06 (1.04-1.08) | <0.001 |

Odds Ratios (ORs) and 95% confidence intervals (CI) for Utstein variables are shown.

© 2025 Nassal MMJ et al. *JAMA Network Open*.

**eTable 3.** Top 3 Survival Quartiles Mixed-Effects Models for Out-of-Hospital Cardiac Arrest Outcomes

| Agency Practice Pattern | ROSC OR (CI) | P-value | Survival OR (95% CI) | P-Value | Survival with favorable neurologic recovery OR (95% CI) | P-Value |
|---|---|---|---|---|---|---|
| Ongoing ETI | 0.80 (0.76,0.83) | 0.000 | 0.88(0.83, 0.94) | 0.000 | 0.84 (0.79, 0.90) | 0.000 |
| Ongoing SGA | 0.71 (0.69, 0.74) | 0.003 | 0.87 (0.83, 0.91) | 0.000 | 0.87 (0.82, 0.91) | 0.000 |
| ETI→SGA | 0.79 (0.76, 0.82) | 0.000 | 0.96 (0.91, 1.01) | 0.167 | 0.94 (0.88, 1.0) | 0.046 |
| SGA→ETI | 0.91 (0.82, 1.02) | 0.115 | 0.85 (0.72, 1.00) | 0.053 | 0.80 (0.67, 0.96) | 0.018 |

Outcomes included return of spontaneous circulation (ROSC), survival to hospital discharge (survival) and survival with favorable neurologic recovery. Odds ratios (OR) are shown with 95% confidence interval (CI).

© 2025 Nassal MMJ et al. *JAMA Network Open*.

**eTable 4.** Bottom Quartile of Unadjusted Out-of-Hospital Cardiac Arrest Outcomes

| Outcomes | Cohort | | Ongoing ETI | | Ongoing SGA | | ETI-to-SGA | | SGA-to-ETI | |
|---|---|---|---|---|---|---|---|---|---|---|
| | Pre 2019 N=28799 | Post 2019 N=35078 | Pre 2019 N=6237 | Post 2019 N=7411 | Pre 2019 N=10686 | Post 2019 N=12961 | Pre 2019 N=7947 | Post 2019 N=9879 | Pre 2019 N=149 | Post 2019 N=114 |
| **ROSC** | 7660 (26.6%) | 8746 (24.9%) | 1919 (30.8%) | 1743 (23.5%) | 2481 (23.2%) | 2762 (21.3%) | 2039 (25.7%) | 2871 (29.1%) | 47 (31.5%) | 32 (28.1%) |
| **Survival to Hospital Discharge** | 1826 / 28698 (6.4%) | 2209 / 34955 (6.3%) | 399 / 6228 (6.4%) | 438 / 7391 (5.9%) | 663 / 10616 (6.3%) | 805/12947 (6.2%) | 442/7929 (5.6%) | 618/9797 (6.3%) | 11/149 (7.4%) | 4/113 (3.5%) |
| **Survival with favorable neurologic recovery** | 1263 (4.4%) | 1518 (4.3%) | 293/6237 (4.7%) | 337/7411 (4.6%) | 418/10688 (3.9%) | 519/12961 (4.0%) | 305/7948 (3.8%) | 401/9879 (4.1%) | 11/149 (7.4%) | 3/114 (2.6%) |

Return of Spontaneous Circulation (ROSC), survival to hospital discharge and survival with favorable neurologic recovery are shown.

© 2025 Nassal MMJ et al. *JAMA Network Open*.
