# Supplementary material for: Advanced Airway Practice Patterns and Out-of-Hospital Cardiac Arrest Outcomes
Source: JAMA Netw Open. 2025 Sep 17;8(9):e2532334. doi: 10.1001/jamanetworkopen.2025.32334 (PMC12444570; doi:10.1001/jamanetworkopen.2025.32334)
Supplement: Supplement 2. — Data Sharing Statement [file jamanetwopen-e2532334-s002.pdf]

Data Sharing Statement

Nassal. Advanced Airway Practice Patterns and Out-of-Hospital Cardiac Arrest Outcomes.
JAMA Netw Open. Published September 17, 2025. doi:10.1001/jamanetworkopen.2025.32334

Data
Data available: Yes
Data types: Deidentified participant data
How to access data: Formal Request from Cardiac Arrest Registry to Enhance Survival
When available: With publication

Supporting Documents
Document types: None

Additional Information
Who can access the data: anyone requesting the data
Types of analyses: for any purpose approved by national CARES
Mechanisms of data availability: After approval of a proposal
